# Catching up missed children and reducing missed opportunities for vaccination in Tanzania: insights from a qualitative analysis paired with an EPI review

**DOI:** 10.11604/pamj.2025.52.141.49151

**Published:** 2025-12-04

**Authors:** William Mwengee, Delphinus Mujuni, Reggis Katsande, Ariel Higgins-Steele, Angela Achieng Omondi, Samuel Bawa, Sarah Wanyoike

**Affiliations:** 1 World Health Organization, Dar es Salaam, Tanzania,; 2 Immunization and Vaccine Development Program, Ministry of Health, Dodoma, Tanzania,; 3 World Health Organization, Regional Office for Africa, Brazzaville, Congo,; 4 World Health Organization, Head Quarter, Geneva, Switzerland,; 5 World Health Organization, Regional Office for Africa, Inter-Country Support Team for East and Southern Africa, Harare, Zimbabwe

**Keywords:** Infant and child vaccination, catch-up, missed children, qualitative analysis, programme strengthening

## Abstract

**Introduction:**

the Expanded Programme on Immunization, initiated by the World Health Organization in 1974, has significantly improved global health by providing equitable access to life-saving vaccines. Despite these achievements, the COVID-19 pandemic caused setbacks in immunization coverage. The Big Catch-Up initiative, launched in April 2023, aims to restore immunization levels by targeting zero-dose and under-immunized children. This study examines BCU integration with routine immunization activities in Tanzania, focusing on the operational dimensions of catch-up efforts, challenges and enabling factors.

**Methods:**

a comprehensive EPI review was conducted in November 2024, incorporating catch-up efforts alongside routine immunization components. Structured BCU questionnaires were developed for four levels of the health system: national, regional, district, and health facility personnel. Data collection involved 66 key informant interviews across various geographic areas. Thematic analysis was used to code and analyze qualitative data, utilizing DelveTool for data management and analysis.

**Results:**

Tanzania's early roll-out of the BCU initiative, combined with polio and measles outbreak responses, demonstrated a proactive approach to addressing immunization gaps. Key strengths included well-articulated goals and targets, catch-up vaccination policy availability, and community engagement. Challenges included limited policy dissemination at lower levels, inconsistent recording practices, and staff shortages. Routinization of catch-up vaccination and capacity-building programmes was identified as essential for sustaining immunization efforts.

**Conclusion:**

integration of catch-up strategies into routine immunization activities and the proactive approach of the Tanzanian government were key success factors. Structured policy dissemination, robust data management systems, and capacity-building programmes are crucial for demonstrating results, sustaining and expanding immunization efforts.

## Introduction

The World Health Organization (WHO) initiated the Expanded Programme on Immunization (EPI) in 1974 to provide all children with equitable access to life-saving vaccines. Since then, EPI has progressed and accomplished significant achievements that transformed the global health scene. It is estimated that vaccinations have prevented 154 million deaths, with 146 million of these among children under 5 years old, including 101 million infants under 1 year; equally averted at least 37 million deaths between 2000 and 2019 [1-3]. However, the COVID-19 pandemic brought about a setback in immunization coverage in many countries [4,5]. The Big Catch-Up (BCU) is a global initiative designed to accelerate immunization coverage by reaching zero-dose and under-immunized children, particularly those left behind due to disruptions caused by the COVID-19 pandemic [6]. Launched in April 2023, the BCU aligns with global immunization strategies and targets, including the Immunization Agenda 2030 and Sustainable Development Goal 3 (Good Health and Well-Being) [7,8]. The BCU initiative aims to restore immunization coverage to pre-pandemic levels, integrate catch-up strategies into routine immunization, and strengthen immunization delivery within primary health care systems to build resilience and sustainability. With urgency to reduce immunity gaps and integrate catch-up strategies into routine immunization activities, an integral part of the BCU initiative is to continuously monitor the process and generate evidence and lessons while the implementation is ongoing [9]. Several assessments are conducted regularly for the EPI to generate information for system strengthening and corrective actions, including Joint Appraisal for Gavi-supported countries, situation analysis, EPI reviews, among others. The WHO recommends periodic comprehensive EPI reviews to identify system-wide barriers, monitor performance against targets, and develop strategic direction for a more robust and resilient programme every 4-5 years [10].

An EPI Review is the comprehensive assessment of the strengths and weaknesses of an immunization programme at national, subnational, and service-delivery levels to prioritize critical programme barriers, highlight programme success, and identify gaps to be addressed [11]. Findings from EPI reviews in East and Southern Africa demonstrated challenges in various aspects of the programmes in different countries, including a lack of NITAGs, poor funding of sessions, and lack of logistics [12]. The United Republic of Tanzania introduced its BCU immunization initiative in 2023, the same year as the global launch. The initiative focused on reducing the number of zero-dose and under-immunized children through a multi-pronged approach that included strengthening routine immunization services, integrating catch-up efforts into outreach activities and supplementary immunization campaigns, and enhancing community engagement. Tanzania´s early efforts to catch up children and reach them for on-time vaccinations yielded results. According to the 2021 WHO/UNICEF Estimates of National Immunization Coverage (WUENIC), Tanzania was ranked number 6 of 21 countries with more than 200,000 zero-dose children, but by 2023, Tanzania´s ranking improved, with the number of estimated zero-dose children reducing to 46,000 [13]. As part of its commitment to strengthening immunization programmes, Tanzania conducted a comprehensive EPI review in November 2024 to assess immunization programme components, including programme management, service delivery, vaccine supply, quality and logistics, surveillance, coverage and monitoring, and demand generation. Recognizing the significant efforts made in 2023-2024 to recover missed vaccinations, a qualitative assessment of BCU implementation was integrated into the review to generate evidence on the operational dimensions of the catch-up activities. The objective is to understand how BCU activities are/were planned, implemented, and identify challenges and enabling factors to inform how catch-up is further institutionalized in the routine system. This study summarizes the qualitative findings on BCU implementation in Tanzania, based on data collected across four levels of the health system during a comprehensive EPI review.

## Methods

**Participant selection and sampling:** a comprehensive EPI review was planned jointly by the WHO, the Ministry of Health (MOH), President's Office, Regional Administration and Local Government Tanzania (PO-RALG), and determined to incorporate catch-up efforts alongside the usual programmatic components of programme management, service delivery, vaccine supply quality and logistics, monitoring and evaluation, and demand generation. A total of 66 participants were purposively selected for the interviews. As shown in Table 1 below, at the national level, two key informants from the MOH for both Mainland Tanzania and Zanzibar were interviewed, ensuring high-level policy perspectives. At the regional level, seven interviews were conducted across a geographically diverse set of regions, encompassing both urban and rural contexts. At the district level, 13 interviews were purposefully held in districts representing multiple regions to reflect variations in district-level implementation and outreach. Finally, at the health facility level, 44 interviews were conducted across a selection of facilities. In total, our purposive sample comprised 66 interviews purposefully selected to provide comprehensive coverage and actionable insights across the system.

**Table 1:** qualitative interviews conducted by health system level and location

Health system level	Interviews (number)	Interview locations
**National**	2	MOH Mainland, MOH Zanzibar
**Region**	7	Dar es Salaam, Kilimanjaro, Lindi, Morogoro, Pwani, Rukwa, Tabora
**District**	13	Biharamulo, Bukoba (Kagera region); Same, Moshi (Kilimanjaro); Nachngwea (Lindi); Mbaral (Mbeya); Morogoro (Morogoro); Njombe (Njombe); Chalinze, Kibaha (Pwani); Kaliua (Tabora); Mjini, Magharibi A (Zanzibar)
**Health facility**	44	5 health facilities in Kagera region; 6 in Kilimanjaro; 4 in Lindi; 6 in Mbeya; 6 in Morogoro; 4 in Njombe; 4 in Pwani; 6 in Tabora; 3 in Zanzibar
**Total**	66	

**Data collection:** structured BCU questionnaires were developed and reviewed jointly by immunization programme specialists from WHO and MoH (WM [PhD, male], DM [PhD, male] and AH-S [PhD, female], SW [MD, female]) experienced in conducting interviews and performing EPI reviews. The questionnaires were specific to four levels of the health system: national, regional, district, and health facility personnel. Questions included semi-structured, open-ended qualitative questions aimed at understanding strengths and areas for improvement related to catch-up efforts and reducing missed opportunities for vaccination, and how to institutionalize catch-up as part of the routine immunization system. Invitations to participate in the EPI review were sent via email through which the investigating team (WM [PhD], DM [PhD], AH-S [PhD], and SW [MD]) provided a thorough explanation of the EPI review mission, objectives, and activities. The BCU questionnaires were administered after the EPI review questionnaire, and the responses were recorded in English on a paper-based data collection tool (either a hard copy or a Word document). Interviews occurred from 15 to 21 November 2024, with each session lasting between 30 and 45 minutes. The research team had no relationship with participants prior to data collection.

**Data analysis:** interview responses were transcribed verbatim from paper-based forms in English and initially consolidated using Microsoft Excel by level administered, e.g. national, region, district, health facility. Following preliminary transcription, we provided participants with concise summaries of their interpreted responses. They were asked to review these interpretations, offer corrections or elaborations, and indicate whether the data accurately reflected their own perspectives. Thematic analysis was used to code and analyze the qualitative data collected. Survey responses were imported into the Delve qualitative analysis tool, where coding and thematic analysis were undertaken. An initial round of open coding was conducted to break down the responses into manageable segments and assign descriptive codes that captured key ideas. Codes were iteratively refined through constant comparison, merging overlapping concepts and distinguishing new patterns as they emerged. These codes were then organized into broader categories, enabling the identification of recurring themes across respondents. Delve´s functionality facilitated systematic coding, clustering of related excerpts, and visualization of emerging patterns, which supported a transparent audit trail of analytic decisions. Through this process, dominant themes and subthemes were generated, reflecting both commonalities and variations in participant perspectives, thereby providing a structured interpretation of the qualitative data.

AAO and AH-S performed the data analysis, and both authors coded and compared transcripts. To enhance credibility, both authors jointly reviewed and deliberated on the content, the labels assigned to codes, and the names of subthemes and themes. Whenever differences arose in how subcategories or categories were coded or labeled, these were resolved through collaborative discussion. The Consolidated Criteria for Reporting Qualitative Research (COREQ) were followed to report the review findings [14].

**Ethics clearance:** conducted as part of the EPI review with the MOH and PO-RALG, the study was part of a programme assessment and obtained all necessary approvals to be undertaken at each level of the health system; however, formal ethics approval was not obtained. Following ethical data collection practices, consent was obtained from respondents, and respondent anonymity was maintained.

## Results

Analysis of the coded survey responses revealed several recurring themes that reflected participants´ experiences and perspectives on the Big Catch-up planning and roll-out across the healthcare system. Themes clustered around frequently quoted catch-up vaccination enabling factors and gaps listed in Table 2 and provide a comprehensive picture of both the strengths that facilitated progress and the persistent obstacles.

**Table 2:** BCU enablers and barriers/gaps themes

Theme Category	Enabling factors	Barriers/Gaps
**Goal and targets**	Well-articulated national goal and target .	Lack of specific BCU targets in a few regions and districts.
**Policy and standard operating procedure (SOP)**	Updated policy and SOP to vaccinate children aged up to 59 months. Targeted dissemination integrated with outbreak response training.	Limited dissemination at lower levels during the early phase; No SOP/catch-up schedule observed at health facilities for delayed doses; broader tailored engagement with non-health system influencers was not done.
**Defaulter tracing / identifying children eligible for catch-up**	Availability of defaulter tracing strategies e.g. house visits by community health workers.	Absence of targeted strategies for tracing older children in a few districts and facilities.
**Data systems and management**	Availability of BCU data collection tools in addition to routine immunization tools e.g. Google sheets, customized reporting tools developed for specific BCU data elements Tracking of additional BCU indicators beyond zero-dose counts and vaccine uptake.	Google Sheets, used as a temporary solution, had limited analytic capabilities and was heavily reliant on internet connectivity; routine and catch-up data integration challenges – technical and administrative hurdles; training gaps – staff turnover limited skills for systems use; inadequate training of healthcare workers on the use of customized tools.
**Stakeholder engagement**	Adequate mapping at subnational levels.	Perception of catch-up as a special activity Inadequate incentive scheme especially for community leaders vaccine hesitancy among some groups.
**Demand generation**	Availability of information, education and communication (IEC) materials targeting diverse stakeholders Strong coordination with local and religious stakeholders The communities work well with community health volunteers.	Community hesitancy especially due to unfamiliarity with vaccination of older children, lack of funds to engage community leaders, limited transportation to distant communities, lack of sensitization for community leaders before activities.
**Routinizing catch-up vaccination**	Advocacy to finance catch-up integration into routine systems Inclusion of catch-up vaccination in the national immunization strategy.	Funding challenges.

Quotes from participants' responses are also presented in Table 3 and Table 3.1 to exemplify each reported theme. A recurring theme among respondents was the importance of having well-defined BCU goals and targets to guide implementation activities, with some districts setting ambitious targets to vaccinate 100% of children who were zero-dose. An updated policy, including catch-up vaccination and SOPs for health workers to use, was developed in 2022 before the rollout of implementation activities in 2023. The policy included revisions to the vaccination schedule to permit vaccination of eligible children under 59 months and was considered supportive by participants. Despite the availability of comprehensive guidelines, respondents at the district and facility levels reported not having hard copies for referencing purposes. The most frequently mentioned enumeration and defaulter tracing strategy was the deployment of CHWs for house-to-house visits for the identification of zero-dose and under-vaccinated children.

**Table 3:** respondent quotes across key thematic areas

Theme	Enablers	Barriers/Gaps
**Goal and targets**	“Close immunity gap caused by missed vaccination particularly during the COVID-19 pandemic. The estimated target was approximately 1.4 million children based on administrative data and community registration methods.” National (Participant 1) “Target of vaccinating 100% zero dose children.” District (Participant 7)	“No specific targets for the BCU." Regional “No specific goals or metrics, just reaching under/un-immunized children (Same).” District (Participant 6)
**Policy and SOP**	“Yes, the policy ensures vaccinations are available to all eligible children including vaccinations up to 59 months for certain vaccines, aligning with routine immunization guidelines.” National (Participant 1) “Yes, there is a policy to vaccinate under five years children.” Regional (Participant 6)	“While there was an effort to disseminate policies within the immediate operational context, broader tailored engagement with influencers outside the health system was not undertaken in the initial phase.” National (Participant 1) “Yes, policy was communicated by letter, but no hard copies were available at district level.” District (Participant 3) “There were no specific BCU guidelines the facilities were aware of. Some facilities did not find any defaulters during their campaigns.” Health Facility (Participant 5)
**Defaulter tracing**	“CHW tools, such as defaulter books, are being modified to include elements for tracking catch-up activities, enabling better follow-up on missed vaccinations at the community level.” National (Participant 1) “The community health worker searches the children from house-to-house and links them to the health facility.” Regional (Participant 1) “CHWs compiled lists of defaulters from registers, reviewed child health cards, or consulted caregivers for eligibility.” Health Facility (Participant 15)	None reported
**Data systems and management**	BCU customized data collection tools “Google Sheets has been beneficial for aggregating data. Google Sheets provided a mechanism to record and report doses administered, including those for children above one year (and above two years for MCV2).” National (Participant 2) b. Tracking additional BCU indicators “Fixed site during working and extended hours, outreach during weekdays and weekends, monitoring during PIRI activities as well.” Regional (Participant 2) “The BCU data collected using excel sheets includes the number of children vaccinated for each antigen, identified zero-dose children, identified defaulters, and total vaccinations. Other key indicators included AEFI, number of fixed sessions, daily percentage and cumulative” District (Participant 9) Training needs “No capacity gaps for them or the councils for tracking BCU data.” Regional (Participant 7)	BCU customized data collection tools “Tally sheet used to record but the tally sheet has gaps of age disaggregation.” Regional (Participant 4) Tracking additional BCU indicators “None” Regional (Participant 3) Training needs “There is a need of training on VIMS/DHIS2 as most of them are new.” District (Participant 12) “There is also not enough staff to conduct BCU activities like outreach and mobile services. The monitoring tools are also insufficient because they do not separate out routine and BCU activities.” Health Facility (Participant 55)

**Table 3.1:** respondent quotes across key thematic areas

Theme	Enablers	Barriers/Gaps
**Stakeholder engagement**	“The community leaders and stakeholders engaged in BCU implementation. However, there was a budget shortage to pay the community leaders. USAID Afya Yangu supported the vaccine transportation, zero effect, etc.” Regional (Participant 8)	“Health workers and stakeholders initially found it challenging to understand and accept the need to vaccinate older age groups beyond the typical focus on children under one year. There was skepticism about the relevance of vaccinating older children with certain vaccines, requiring capacity building to address this.” National, (Participant 2) “Lack of dedicated funds to motivate community leaders to fully participate in BCU activities.” Regional (Participant 4) “Inadequate funds to support community leaders during mobilization.” District (Participant 13)
**Demand generation**	“The community leaders and stakeholders engaged in BCU implementation.” Regional (Participant 8) “Meetings were held with caretakers to mobilize them and with CHWs as well.” Health Facility (Participant 43)	“Engaging religious leaders in demand-creation activities can be challenging. Often, only those who are already supportive participate—while those who are hesitant or opposed may remain unengaged. This limits our ability to understand and address their concerns.” National (Participant 2) “Facilities reported hesitancy, where vaccinating older children was unfamiliar to the community.” Health Facility (Participant 16)
**Routinizing catch-up vaccination**	“Catch-up vaccination is clearly outlined in the national immunization strategy.” National (Participant 1) “Yes, included in the RHMT Plan.” Regional (Participant 7) “The plan is to continue these activities; however, as they will now need to budget for them, they need to find additional funding." District (Participant 1)	“Significant funding gaps, particularly for emergent activities like community-level mobilization and vaccination during intensification exercises. Routine vaccine doses are being used to cover catch-up needs, which risks creating shortages.” National (Participant 1)

Regarding BCU data collection systems, Google Sheets, which was deployed as an interim solution to collect catch-up data, was considered beneficial by most respondents across all levels of the health system. Despite its usefulness, issues with age disaggregation, challenges with internet connectivity, manual consolidation processes that cause delays, and training needs due to limited analytical capabilities were reported. Additionally, the Afya Campaign Data Management System was also used to capture campaign data from the facility level. Existing tools, such as tally sheets, were also revised to allow for the recording and reporting of extended age group data, with age disaggregation into <1 year and >1 year. BCU monitoring also included the use of additional indicators beyond zero-dose counts and vaccine utilization data, such as service delivery modalities and the identification of hard-to-reach areas for outreach.

While Tanzania´s BCU activities were reported to engage key community stakeholders, the absence of incentive schemes in most regions and districts emerged as a significant barrier in mobilizing community leaders for identifying zero-dose and under-vaccinated children and promoting catch-up vaccination. Additionally, vaccine hesitancy among certain religious leaders, compounded by a general unfamiliarity with older-child vaccination, further undermined demand-generation efforts.

Respondents acknowledged the importance of sustaining catch-up vaccination beyond the BCU initiative, primarily through integration into the routine immunization system by including catch-up in national and regional planning. adaptation of data collection tools to record and report extended ages, leveraging periodic intensification of routine immunization (PIRI) to catch-up older children. However, routinized catch-up vaccination was contingent upon securing additional funding to support the added operational demands. Overall, these results clearly emphasize the importance of resource allocation, training, and community sensitization to overcome persistent barriers.

## Discussion

The study demonstrates that integrating the BCU initiative assessment within an EPI review is possible and can enable a comprehensive assessment of both routine and catch-up immunization efforts. This approach optimized time and resources while ensuring a thorough evaluation of immunization activities. We found that Tanzania´s MOH and PO-RALG mobilized an early roll-out of the catch-up strategy compared to other countries globally, combining response to a circulating vaccine-derived virus type 2 (cVDPV2) and measles outbreaks, to reach children missing multiple vaccine doses. This enhanced acceleration of the BCU initiative without delays, using vaccine supply available in-country, which was subsequently replenished. This is similar to studies reported from Nigeria and Nepal, which demonstrate the centrality of political will and ownership in the success of immunization initiatives to achieve the Sustainable Development Goals [15].

The early roll-out of catch-up efforts in Tanzania was aggravated by the polio outbreak due to the importation of cVDPV2 [16]. The country leveraged the opportunity to mount efforts at improving population immunity against other vaccine-preventable diseases. This corroborates with the SOPs of polio outbreak response, where it is used as an opportunity to strengthen routine immunization [17]. Furthermore, it demonstrates the effectiveness of integration, where resources are leveraged to address multiple interventions [18,19].

The availability of an updated national policy framework with extended eligibility for catch-up doses to children up to 59 months is a critical success determinant for large-scale immunization initiatives, such as the BCU. It serves as the implementation blueprint guiding frontline workers and immunization managers in delivering services uniformly with age thresholds and minimum intervals between vaccine doses, thereby avoiding ambiguity and inconsistent practices. Additionally, a BCU policy framework safeguards against the loss of older children from vaccination efforts. As children grow older, they are less likely to be reached through routine channels, and without an explicit policy, a child missing on-time vaccine doses risks being overlooked. Maintaining eligibility to vaccinate older children ensures continued opportunities for protection and follows WHO´s recommendations for interrupted or delayed routine immunization [20]. Policy dissemination in Tanzania´s experience was primarily health system-focused and initially context-specific, implemented alongside outbreak response activities prioritizing measles-affected regions first, followed by a phased roll-out to other regions. Early dissemination efforts focused on orienting health workers in applying the updated schedules for delayed or interrupted vaccinations. While district and health facility teams were generally aware of the updated policy and associated catch-up schedule and SOPs, hard copies were unavailable in some facilities, and this may have negatively impacted implementation efforts.

Defaulter or drop-out tracing is an integral catch-up vaccination activity to identify and enumerate eligible children and heavily relies on community volunteers. Frequently reported strategies were review of the immunization registry and use of CHWs for house-to-house identification and referral to health facilities. CHWs are particularly valuable as they often have firsthand knowledge of areas with children who missed vaccination and underserved communities within their communities and can effectively collaborate with community leaders to facilitate access to these children and locations. Other strategies at the facility level included screening children during RCH visits, leveraging outreach sessions to identify children who missed vaccination, strengthening post-natal mother follow-up, providing health education at churches and mosques, and increasing caregiver awareness of catch-up eligibility screening to reduce missed vaccination opportunities. These findings underscore the importance of interventions that enhance engagement between health care workers and caregivers to promote health education, the importance of timely routine immunization in ensuring optimal protection to enhance defaulter tracing, and ultimately reduce missed opportunities for vaccination [21-23].

The deployment of multiple data collection systems adapted to capture age-disaggregated data (<1 year and >1 year) facilitated structured monitoring of catch-up vaccination performance. However, reliance on disparate systems introduced significant challenges. Google Sheets, while flexible and accessible for quick deployment, lacked robust offline functionality, version control, and built-in validation features, which raised the risks of duplicate entries and inconsistent data formats. Manual consolidation of data from multiple tools, combined with insufficient training of healthcare workers on customized tools, further amplified the potential for error and undermined data quality and consistency. The fragmented use of systems also complicated smooth integration into the national routine platform, DHIS2. While integration of catch-up indicators into DHIS2 was underway, an effort aimed at institutionalizing age-disaggregated reporting and enhancing sustainability the transitional period affected data reporting timeliness. Published reviews emphasize that while multiple immunization data collection systems can enable flexibility, they also exacerbate common health data challenges in low- and middle-income countries, such as fragmentation, limited interoperability, and inconsistent data quality [24,25]. In summary, while using various tools provided short-term functionality and flexibility for the catch-up campaign, long-term success hinges on fully integrating these data streams, particularly age-disaggregated catch-up indicators into centralized, well-supported platforms, such as DHIS2. Investments in infrastructure, user training, and interoperability are crucial for enhancing the quality, consistency, and sustainability of immunization monitoring efforts [26,27].

BCU monitoring efforts were enhanced by additional indicators enabling assessment of catch-up vaccination beyond zero-dose performance and vaccine utilization monitoring. Relying only on the number of ZD children vaccinated risks underestimating coverage gains, overlooking operational bottlenecks, and missing opportunities for programme improvement. Tracking multiple indicators supports evidence-based planning, resource allocation, and informed policy decisions for achieving and sustaining immunization equity. Additional indicators encompassing percentage of children vaccinated by different service delivery modalities; dropout rates; CHW effectiveness in identifying defaulters during house-to-house visits and outreach campaigns; tracking of BCU-specific data integration into VIMS and DHIS2 routine systems for consistency; monitoring vaccine logistics beyond uptake, including the availability of additional doses and stockouts; accessibility of immunization services at health facilities were all leveraged across the system to monitor BCU progress.

Stakeholder engagement and demand generation strategies varied across the health system. At the national level, coordination between the MoH, WHO, and UNICEF facilitated policy alignment and resource mobilization. At subnational levels, CHWs, religious leaders, and other community influencers were instrumental in promoting vaccine uptake. CHWs and religious leaders played a crucial role in increasing vaccine acceptance and uptake through community mobilization and awareness campaigns. This corroborates published findings on the critical role of CHWs and community leaders in improving vaccine uptake [28-30]. However, the limited engagement of private healthcare providers, schools, and local government authorities constrained reach in some districts. Furthermore, limited funding for community engagement also impacted demand for vaccination. Strengthening multi-sectoral collaboration, ensuring consistent advocacy messaging, and securing dedicated budgets for outreach activities could enhance both coverage and sustainability.

The BCUs' three-pronged framework, catch-up, restore, and strengthen programme, was designed not only to close immediate coverage gaps but also to provide a pathway toward institutionalizing catch-up vaccination into the routine immunization system. Tanzania´s BCU routinization efforts were enabled by the inclusion of catch-up vaccination into national immunization policies and subnational plans, the adaptation of data collection systems to record and report extended age groups, and health workforce training to identify zero-dose and under-vaccinated children. Respondent feedback emphasizes that while structural integration and strategic intensification are promising pathways, their success depends critically on sustained financing. Bridging these funding gaps is crucial to fully harness the potential of integrated catch-up strategies and prevent compromising routine service delivery.

**Limitations:** this study was not without limitations. As this was not a standalone qualitative study and while benefiting from the data collection opportunity of an EPI review, combining data collection exercises can lead to longer interviews and respondent fatigue. However, the research interview was conducted by engaging in interpersonal communication skills for efficient interview sessions. Paper-based tools were used for data collection without audio recording, which meant that some nuance in respondent answers may not have been captured.

## Conclusion

The study highlights the importance of a structured approach to implementing catch-up immunization policy dissemination, robust data management systems, and capacity-building programmes to sustain and expand immunization efforts, thereby reaching children who are missing vaccine doses. The integration of catch-up strategies into routine immunization activities and the proactive approach of the Tanzanian government were key factors in the success of the BCU initiative.

### What is known about this topic

- EPI reviews and immunization programme assessments are recommended and conducted in countries every few years;
- Routine immunization traditionally focused on reaching children under two years of age;
- COVID-19 pandemic led to disruptions in health services and backsliding of immunization coverage.

### What this study adds

- Integration of new or priority immunization topics can be included in assessments, reviews, and/or surveys save time and resources;
- Political willingness and proactiveness to restore immunization system and close immunity gaps in the context of a major disruption;
- Adaptation of policies and structured roll-out facilitates health system responsiveness to vaccinate infants on time, as well as catch-up children more than 2 years of age who are missing RI doses.

## Available online at

https://www.panafrican-med-journal.com/content/article/52/141/full

## References

[1] Shattock AJ, Johnson HC, Sim SY, Carter A, Lambach P, Hutubessy RCW (2024). Contribution of vaccination to improved survival and health: modelling 50 years of the Expanded Programme on Immunization. The Lancet.

[2] Lindstrand A, Cherian T, Chang-Blanc D, Feikin D, O´Brien KL (2021). The World of Immunization: Achievements, Challenges, and Strategic Vision for the Next Decade. The Journal of Infectious Diseases.

[3] Vashishtha VM, Kumar P (2013). 50 years of immunization in India: Progress and future. Indian Pediatr.

[4] Lassi ZS, Naseem R, Salam RA, Siddiqui F, Das JK (2021). The Impact of the COVID-19 Pandemic on Immunization Campaigns and Programs: A Systematic Review. IJERPH.

[5] Basu S, Ashok G, Debroy R, Ramaiah S, Livingstone P, Anbarasu A (2023). Impact of the COVID-19 pandemic on routine vaccine landscape: A global perspective. Human Vaccines & Immunotherapeutics.

[6] World Health Organization The Big Catch-Up: An Essential Immunization Recovery Plan for 2023 and Beyond.

[7] World Health Organization Global Vaccine Action Plan Monitoring, Evaluation & Accountability: Secretariat Annual Report 2020.

[8] Immunization Agenda 2030 Partners (2024). Immunization Agenda 2030: A global strategy to leave no one behind. Vaccine.

[9] Lindstrand A, Mast E, Churchill S, Rahimi N, Grevendork J, Brooks A (2024). Implementing the immunization agenda 2030: A framework for action through coordinated planning, monitoring & evaluation, ownership & accountability, and communications & advocacy. Vaccine.

[10] World Health Organization (2017). A guide for conducting an Expanded Programme on Immunization (EPI) Review.

[11] Lyimo D, Kamugisha C, Yohana E, Shibehsi ME, Wallace A, Ward K (2017). Improving the efficiency and standards of a National Immunization Program Review: lessons learnt from United Republic of Tanzania. Pan Afr Med J.

[12] Shibeshi ME, Masresha BG, Daniel F (2021). Immunisation program reviews in East and Southern Africa. 2012-2018; key lessons. Pan Afr Med J.

[13] Sangeda RZ, James D, Mariki H, Mbwambo ME, Mwenesi ME, Nyaki H (2024). Childhood vaccination trends during 2019 to 2022 in Tanzania and the impact of the COVID-19 pandemic. Human Vaccines & Immunotherapeutics.

[14] Tong A, Sainsbury P, Craig J (2007). Consolidated criteria for reporting qualitative research (COREQ): a 32-item checklist for interviews and focus groups. International Journal for Quality in Health Care.

[15] Wonodi C, Crowcroft N, Bose AS, Oteri J, Momoh J, Hughes G (2024). Country ownership as a guiding principle for IA2030: A case study of the measles and rubella elimination programs in Nepal and Nigeria. Vaccine.

[16] World Health Organization (2023). Circulating vaccine-derived poliovirus type 2 (cVDPV2) - United Republic of Tanzania.

[17] World Health Organization (2022). Standard operating procedures: responding to a poliovirus event or outbreak, version 4.

[18] Anya BM, Moturi E, Aschalew T, Carole Tevi-Benissan M, Akanmori BD, Poy AN (2016). Contribution of polio eradication initiative to strengthening routine immunization: Lessons learnt in the WHO African region. Vaccine.

[19] Dudley L, Garner P (2011). Strategies for integrating primary health services in low-and middle-income countries at the point of delivery. Cochrane Database of Systematic Reviews.

[20] World Health Organization (2024). Table 3 Recommendations for Interrupted or Delayed Routine Immunization (updated: December 2024). WHO.

[21] Bakkabulindi P, Ampeire I, Ayebale L, Mubiri P, Feletto M, Muhumuza S (2023). Engagement of community health workers to improve immunization coverage through addressing inequities and enhancing data quality and use is a feasible and effective approach: An implementation study in Uganda. PLoS ONE.

[22] Dhaliwal BK, Mathew JL, Obiagwu PN, Hill R, Wonodi CB, Best T (2024). Addressing Missed Opportunities for Vaccination among Children in Hospitals: Leveraging the P-Process for Health Communication Strategies. Vaccines.

[23] Fallucca A, Priano W, Carubia A, Ferro P, Pisciotta V, Casuccio A (2024). Effectiveness of Catch-Up Vaccination Interventions Versus Standard or Usual Care Procedures in Increasing Adherence to Recommended Vaccinations Among Different Age Groups: Systematic Review and Meta-Analysis of Randomized Controlled Trials and Before-After Studies. JMIR Public Health Surveill.

[24] Rahmadhan MAWP, Handayani PW (2023). Challenges of vaccination information system implementation: A systematic literature review. Human Vaccines & Immunotherapeutics.

[25] Harrison K, Rahimi N, Carolina Danovaro-Holliday M (2020). Factors limiting data quality in the expanded programme on immunization in low and middle-income countries: A scoping review. Vaccine.

[26] Scobie HM, Edelstein M, Nicol E, Morice A, Rahimi N, MacDonald NE (2020). Improving the quality and use of immunization and surveillance data: Summary report of the Working Group of the Strategic Advisory Group of Experts on Immunization. Vaccine.

[27] Pavia G, Branda F, Ciccozzi A, Romano C, Locci C, Azzena I (2024). Integrating Digital Health Solutions with Immunization Strategies: Improving Immunization Coverage and Monitoring in the Post-COVID-19 Era. Vaccines.

[28] Nzioki JM, Ouma J, Ombaka JH, Onyango RO (2017). Community health worker interventions are key to optimal infant immunization coverage, evidence from a pretest-posttest experiment in Mwingi, Kenya. Pan Afr Med J.

[29] Mohammed Y, Reynolds HW, Waziri H, Attahiru A, Olowo-okere A, Kamateeka M (2024). Exploring the landscape of routine immunization in Nigeria: A scoping review of barriers and facilitators. Vaccine: X.

[30] Habib MA, Soofi S, Cousens S, Anwar S, Haque NU, Ahmed I (2017). Community engagement and integrated health and polio immunisation campaigns in conflict-affected areas of Pakistan: a cluster randomised controlled trial. The Lancet Global Health.

